# Real-time FPGA-based implementation of the AKAZE algorithm with nonlinear scale space generation using image partitioning

**DOI:** 10.1007/s11554-021-01089-9

**Published:** 2021-03-29

**Authors:** Parastoo Soleimani, David W. Capson, Kin Fun Li

**Affiliations:** Department of Electrical and Computer Engineering, University of Victoria, Victoria, BC V8W 2Y2 Canada

**Keywords:** AKAZE, FPGA, Nonlinear scale space, Hardware design, Real-time, Image matching

## Abstract

The first step in a scale invariant image matching system is scale space generation. Nonlinear scale space generation algorithms such as AKAZE, reduce noise and distortion in different scales while retaining the borders and key-points of the image. An FPGA-based hardware architecture for AKAZE nonlinear scale space generation is proposed to speed up this algorithm for real-time applications. The three contributions of this work are (1) mapping the two passes of the AKAZE algorithm onto a hardware architecture that realizes parallel processing of multiple sections, (2) multi-scale line buffers which can be used for different scales, and (3) a time-sharing mechanism in the memory management unit to process multiple sections of the image in parallel. We propose a time-sharing mechanism for memory management to prevent artifacts as a result of separating the process of image partitioning. We also use approximations in the algorithm to make hardware implementation more efficient while maintaining the repeatability of the detection. A frame rate of 304 frames per second for a $1280 \times 768$ image resolution is achieved which is favorably faster in comparison with other work.

## Introduction

Feature detection and description are two of the important stages in many computer vision algorithms such as object recognition, face recognition, image stitching, image retrieval, camera localization, and so on. One important criterion in choosing a feature detector is having high repeatability. Repeatability is defined as the capability of finding the same feature in different viewpoints and scales. In feature detection, repeatable points of interest in the image are detected, and in feature description, for each detected point, a descriptor is defined to be matched to the same key-point in other images. An important characteristic of a feature detector is invariance to scale changes.

Scale invariant feature transform (SIFT) [1] and speeded up robust features (SURF) [2] are two popular multi-scale feature detector and descriptor algorithms. Both approaches are computationally expensive. Oriented FAST and rotated BRIEF (ORB) [3] and binary robust invariant scalable keypoints (BRISK) [4] feature detector and descriptor algorithms were introduced to reduce the computational time of the matching algorithm and to increase speed by using the features from accelerated segment test (FAST) [5] detector and binary robust independent elementary features (BRIEF) [6] based binary descriptors.

The KAZE [7] feature detector and descriptor is another multi-scale approach that uses nonlinear filtering instead of a Gaussian filter, to create scale space and achieve improvement in terms of repeatability in comparison with other approaches. The main drawback of the KAZE feature detector and descriptor is its speed in comparison with other approaches, which is due to the nonlinear scale space. The accelerated KAZE (AKAZE) [8] approach was introduced to speed up the KAZE algorithm by using a mathematical framework called fast explicit diffusion (FED) to build a nonlinear scale space, and by introducing a new descriptor named modified local difference binary (M-LDB) to reduce storage requirement. Although it has been demonstrated in the original AKAZE paper [8] that this algorithm outperforms other algorithms such as SIFT, SURF, ORB, and BRISK in terms of repeatability and accuracy, it is still slower in comparison with ORB and BRISK due to the nonlinear scale space creation.

As the demand for embedded vision systems has been increasing in recent years, implementing real-time algorithms while maintaining accuracy has become more important. Although the AKAZE algorithm is less computationally expensive in comparison with the KAZE algorithm due to the FED filters, it still has higher computational complexity compared to ORB and BRISK detectors. There are many attempts for implementations of image processing and other algorithms using Field Programmable Gate Array (FPGA) due to its parallel architecture and speed benefits [9–11]. In this paper an FPGA-based accelerator for the AKAZE feature detector is introduced to achieve higher speed while keeping the same repeatability as the original AKAZE.

## Related work

The SIFT feature detector and descriptor was introduced in 2004 and is based on the difference of Gaussians (DoG) operator. The detector is applied at different scales of an image and for each detected key-point, a $16 \times 16$ s patch is extracted and segmented into 16 sub regions. For each sub region, a histogram of gradients is generated. The descriptor is the concatenation of these histograms. The main drawback of SIFT is its computational cost.

To reduce the computational cost of SIFT, SURF was introduced in 2008. SURF uses the determinant of a Hessian matrix in its detector and takes advantage of integral images to increase the speed of the detection. For each detected key-point, the descriptor is defined by using Haar wavelet responses of its surrounding patch. In 2011, ORB was introduced. ORB uses FAST as a detector and a modified version of BRIEF as its descriptor.

The KAZE algorithm was introduced in 2012 using non-linear scale space. The detector used in KAZE is based on the determinant of a Hessian matrix and the descriptor is based on the local difference binary (LDB) descriptor. By using non-linear diffusion filtering, the boundaries of the regions in different scales are retained, while reducing noise in the image. Other previous methods find features using a Gaussian scale space which smooths noise and boundaries of objects to the same degree which results in the loss of detail. The KAZE algorithm is rotation-invariant and scale-invariant, and has more distinctiveness at various scales, but it is slower in comparison with other algorithms.

To overcome this drawback, the accelerated KAZE (AKAZE) algorithm was proposed in 2013. AKAZE non-linear diffusion filtering is based on a fast explicit diffusion (FED) framework which is more efficient in comparison with KAZE filtering. The AKAZE detector is based on the determinant of a Hessian matrix and the AKAZE descriptor is the modified local difference binary (MLDB). Although AKAZE is faster in comparison with the KAZE algorithm, it is still slower than binary descriptors such as ORB and BRISK. In this work we propose a hardware design to accelerate the AKAZE algorithm.

There are multiple publications that propose accelerators for the AKAZE algorithm. Ramkumar et al. [12] propose a GPU-based implementation of the KAZE algorithm. Jiang et al. [13] describe a hardware architecture for the AKAZE algorithm based on application specific integrated circuits. They achieve a throughput of 127 frames per second for $1920 \times 1080$ images. However, their design does not cover the contrast factor calculation which is an essential part of the AKAZE algorithm. The AKAZE algorithm requires two passes through the image and by not implementing the contrast factor, they are eliminating one of the passes which contributes to higher throughput.

Kalms et al. [14] introduce a hardware accelerator based on FPGAs for extracting AKAZE features. In their initial publication, they propose a pipelined architecture for nonlinear scale space generation and they assume that the contrast factor is computed in software. In their later work [15], they design an architecture for contrast factor computation as well. They achieve a frame rate of 98 frames per second for a $1024 \times 768$ image resolution.

Mentzer et al. [16] propose a hardware accelerator for the AKAZE algorithm based on application specific instruction-set processors (ASIP) which is used for an advanced driving assistance system. They achieve a frame rate of 20 frames per second which is higher than the results obtained from a conventional processor and consumes less power than the FPGAs.

Li et al. [17] use the AKAZE algorithm for extracting descriptors from a video sequence. They use previous frame pixels to predict the first octave of the nonlinear scale space of the current frame in the AKAZE algorithm to increase speed. They achieve 784 frames per second for $640 \times 480$ images. They propose using motion estimation to reduce the effect of using the previous frame. Still, based on the results they published, this method decreases the accuracy of the algorithm. Their method is beneficial in applications which process high video frame rates in which the amount of changes in successive frames is negligible.

In this work, we take advantage of the fact that the algorithm uses two passes through the input image. For the first pass, we read the image and store it on the FPGA. In the second pass, we process the image in parallel to achieve increased speed. In comparison with [17], our method does not require the previous frames to process the current frame. We achieve a higher frame rate than [15] at the same image resolution and frequency by introducing a memory management unit which facilitates the parallel processing of the image.

## A brief introduction to AKAZE nonlinear scale space generation

**Fig. 1:**
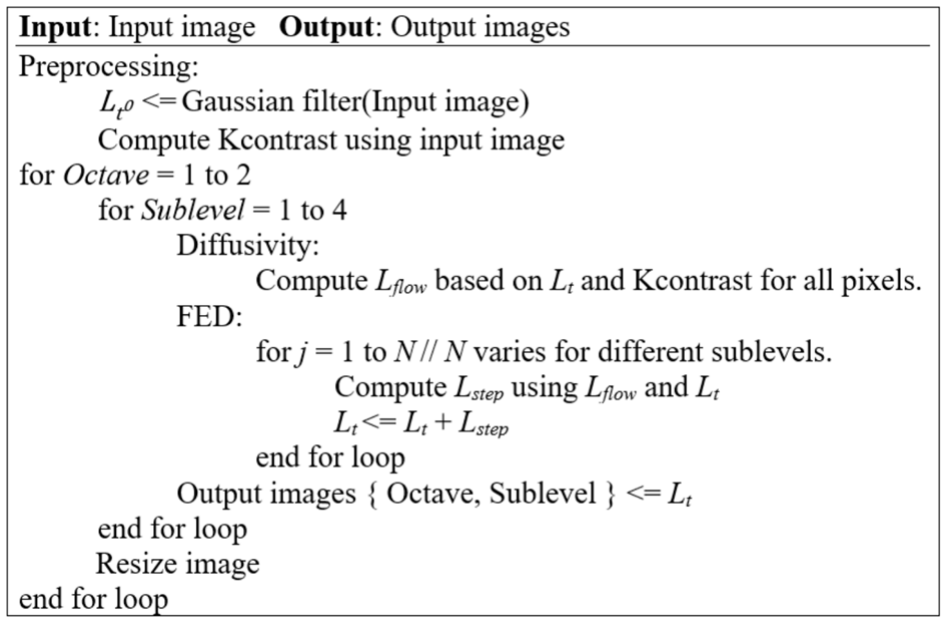
Pseudocode of AKAZE algorithm

The nonlinear scale space is a set of different scales of the input image. These scales are grouped as octaves which each of them having four sublevels in the AKAZE algorithm. Figure 1 shows a pseudocode overview of the algorithm for two octaves.

**Fig. 2:**
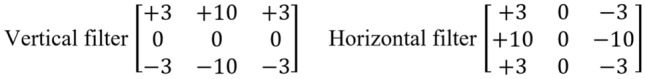
Scharr filter weights

The preprocessing step of the AKAZE algorithm generates a nonlinear scale space. In this step, the image is Gaussian filtered to reduce noise. Then, since the contrast of the image has significant effects on extracting the details of the image, a contrast factor is computed (for use in subsequent steps). In the second step, which computes diffusivity, a conductivity function [8] is calculated using image gradients and a contrast factor found in the preprocessing step. This function affects how much detail of the boundaries of the image is retained in the filtering process. In this work, we use the conductivity function [8] in Eq. (1), as follows:

1 $$\begin{aligned} L_\mathrm{flow} (i,j)=\frac{1}{1+\frac{L^2_x(i,j)+L^2_y(i,j)}{K^2}} \end{aligned}$$

where *K* is the contrast factor and $L_{x}$ and $L_{y}$ are the gradients of the image computed using a Scharr filter in horizontal and vertical directions, respectively. We use the Scharr filter parameters as shown in Fig. 2.

The output of the diffusivity step is called $L_\mathrm{flow}$ which is computed for each pixel of the image. In the third and final step, which computes the FED, the new sublevel scale is generated using $L_\mathrm{flow}$ and the previous sublevel. The FED process has multiple iterations (*N*), the number of which varies depending on the level of the scale space. The value of (*N*) for each sublevel is determined using a precomputed array from the original AKAZE algorithm [8]. In each step, a constant step size value is multiplied by the filter.

In each FED process, the summation of the center pixel with four adjacent pixels in vertical and horizontal directions of $L_\mathrm{flow}$ are multiplied by the difference between the center pixel with four adjacent pixels in vertical and horizontal directions of the previous sublevel. The summation of the results of the multiplications is called $L_\mathrm{step}$. The FED calculations are shown in Eqs. (2) and (3):

2 $$\begin{aligned}&L_\mathrm{step} (i,j) =\Sigma (L_\mathrm{flow}(i,j)+L_\mathrm{flow}(i+k_1,j+k_2))+\nonumber \\&(L_{t^n} (i,j)-L_{t^n} (i+k_1,j+k_2))s \end{aligned}$$

with $k_1, k_2 \in \{-1,1\}$ where $L_\mathrm{step}$ is the output of the FED calculation, $L_{t}$ is the previous sublevel and *s* is the step size constant which is different for each sublevel. The next sublevel is generated as given in Eq. (3):

3 $$\begin{aligned} L_{t^{n+1}}=L_\mathrm{step}+L_{t^n} \end{aligned}$$

where $L_{t^{n+1}}$ is the value of the next sublevel in the nonlinear scale space.

## Hardware implementation

**Fig. 3:**
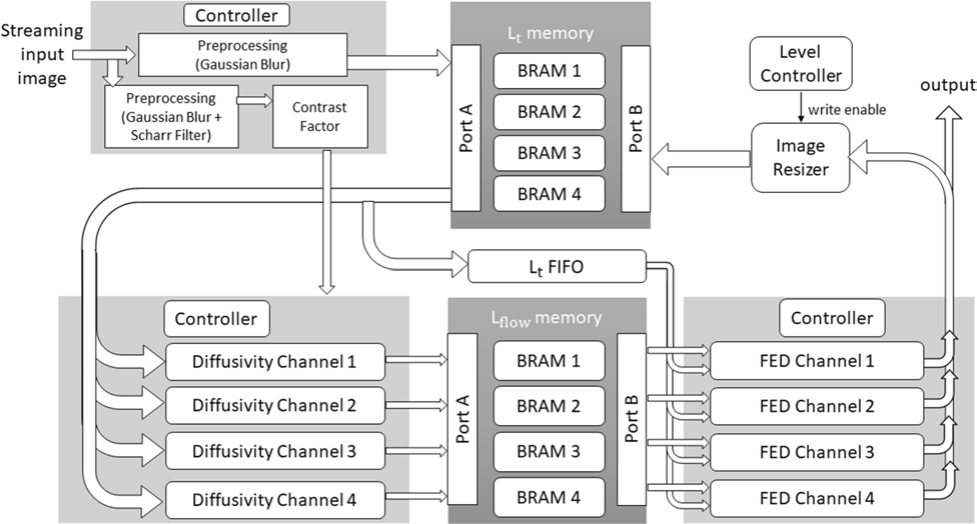
Block diagram of AKAZE scale space generation with four channels

Figure 3 is the overall block diagram of AKAZE scale space generation. The main contribution of this work is based on the fact that this algorithm has two passes through the input data. We take advantage of this fact by storing the data in the first pass and process it in parallel in the second pass. We need two memory units for storing the sublevels ($L_{t}$) and the output of the conductivity function ($L_\mathrm{flow}$). Each of these two memories has the capacity to store a full image. These two memories are implemented in the Block RAMs (BRAM) of the FPGA. Each BRAM comprises a group of four smaller BRAMs which store a section of an image, divided vertically. The first set of BRAMs contains $L_{t}$ data and the second set of BRAMs stores $L_\mathrm{flow}$ data.

This design has three stages. In the first stage (the preprocessing stage), the 8-bit grey level image enters pixel by pixel to the preprocessing unit in which the contrast factor of the image is calculated, and the image is filtered using a Gaussian blur filter. The contrast factor value is used further in the diffusivity unit, which is the second stage of this design. Then, we store the filtered image, which is the first level of the nonlinear scale space, in $L_{t}$ memory.

**Fig. 4:**
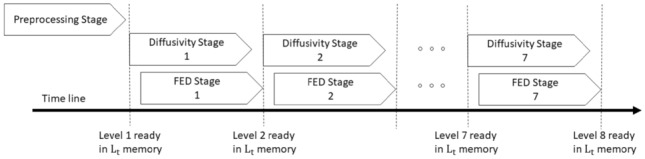
Data flow of the algorithm. The FED stage starts after the diffusivity stage. The preprocessing stage only processes the data once at the beginning of the algorithm while the diffusivity and FED stages run in each iteration

After first stage is completed, the second stage (the diffusivity unit) begins. This unit stores the values in $L_\mathrm{flow}$ memory in preparation for the third stage, which is FED calculation. From there on, stage 2 and stage 3 work simultaneously until all sublevels are generated. The output of the third stage is the sublevels of the nonlinear scale space which are written back to $L_{t}$ memory for the next iteration. Figure 4 shows the data flow of the algorithm at all stages. Further details of each stage are explained in the following sections.

### Stage 1: the preprocessing unit

**Fig. 5:**
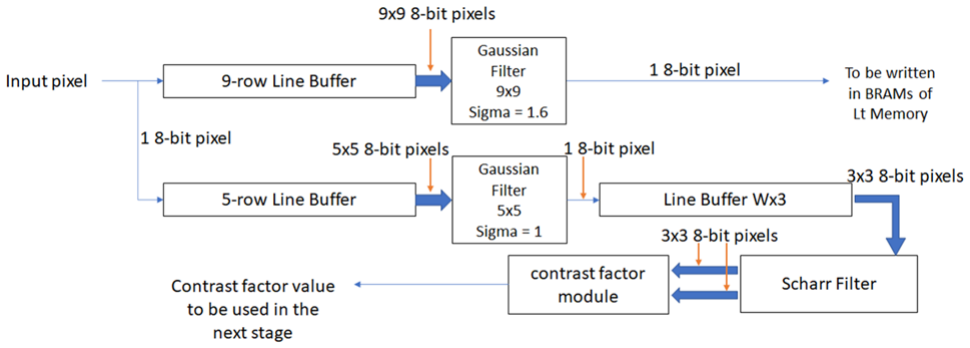
Preprocessing stage architecture. This stage contains two Gaussian filter modules. The output of the 9-row line buffer is a  9 × 9 window and the output of the 5-row line buffer is a 5 × 5 window. This stage computes the contrast factor and stores the filtered image in the memory

The block diagram of the preprocessing unit is shown in Fig. 5. This unit has two outputs. The first output is the filtered image, which is the first sublevel and initial value of $L_{t}$, and is stored in the $L_{t}$ BRAMs. The second output of this unit is the contrast factor of the image, which is used in Stage 2 for the calculation of image diffusivity. To calculate the first sublevel, a $9 \times 9$ Gaussian filter is required. The image first enters a line buffer that has a size of $W \times 9$, where *W* is the image width. The $9 \times 9$ window at the end of the line buffer is connected to a Gaussian filter module, in which the filtered value for the center pixel in the $9 \times 9$ window is calculated and is stored in the corresponding $L_{t}$ BRAM memory.

To calculate the contrast factor, first, we apply a $5 \times 5$ Gaussian filter to the image. The architecture for this filter is similar to a $9 \times 9$ filter and differs only in the size of line buffer and filter module. After filtering the image, the gradients of the image in horizontal and vertical directions are calculated using Scharr filters. The outputs of the Scharr filters are used by the contrast factor calculation module. Finally, the result of the contrast factor module is sent to the diffusivity calculation unit which is the next stage of the algorithm.

**Fig. 6:**
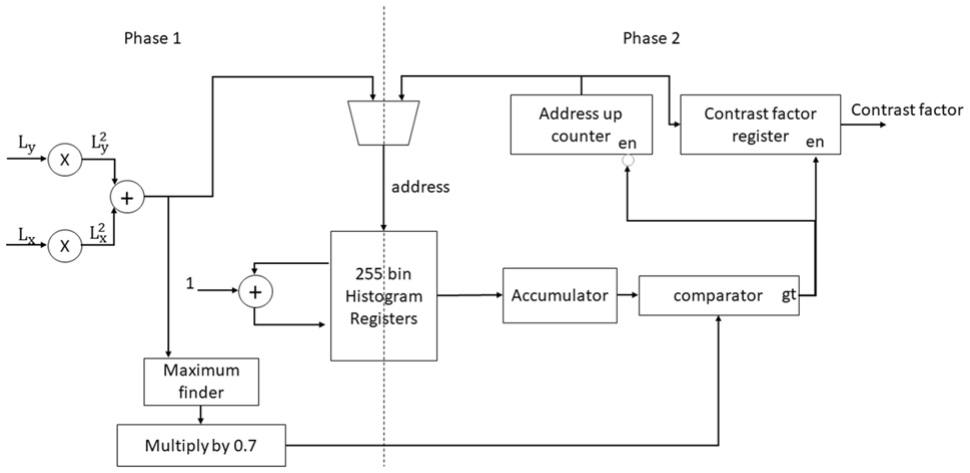
Block diagram of contrast factor calculation module

The block diagram of the contrast factor module is shown in Fig. 6. This module receives the horizontal and vertical gradients as input and generates the value of the contrast factor as output. The process of computing the contrast factor value has two phases which is shown in Fig. 6. In the first phase, the value of $L_x^2+L_y^2$ is computed. In the original algorithm, the square root of $L_x^2+L_y^2$ is used. However, since this value is used as an address for histogram generation, we can safely set aside the square root. We map this value to 0 to 255 by normalization. This value is used as the address of a set of 256 registers storing the histogram. At each clock cycle, we increment the value of the corresponding register to which $L_x^2+L_y^2$ is pointing. At the same time, we store the maximum of this value in the maximum finder register. After this step is finished and the histogram is built, in the second phase, we start from the beginning of the histogram and read the values of the registers and add them in the accumulator. Whenever the value in the accumulator reaches 70% of the maximum value of $L_x^2+L_y^2$ from phase 1, we store the bin number (same as address value) in the contrast factor register. The value in the contrast factor register is the output of the module.

### Stage 2: diffusivity calculation

**Fig. 7:**
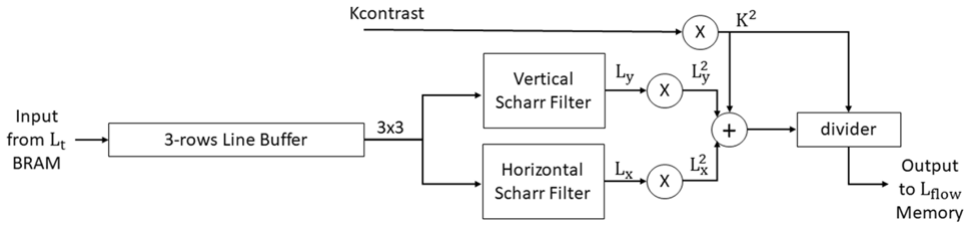
Diffusivity channel architecture

After storing the first sublevel $L_{t^{0}}$ in the $L_{t}$ memories in the first stage, the second stage, which is the diffusivity stage, begins. Figure 7 shows the architecture of a diffusivity channel. In this stage, we read the data from the $L_{t}$ BRAMs, and the contrast factor value of the image. The contrast factor value is fixed for each image and does not change in the next steps of the algorithm. The $L_{t}$ data which we read from the BRAM memory enter a 3-row line buffer. The output of the line buffer is connected to two Scharr filters. We compute the gradients of $L_{t}$ data in x-direction and y-direction using Scharr filters and label them as $L_{x}$ and $L_{y}$, respectively. Then, by using $L_{x}$ value and $L_{y}$ value and the contrast factor, we compute the value of $L_\mathrm{flow}$ according to Eq. (4). For computing $L_\mathrm{flow}$, we use a divider IP core provided by *Xilinx*^®^ [18] which has 43 clock cycles delay. The divisor and the dividend inputs of the IP core are 24-bit and 16-bit integers, respectively. The output of the divider is a fixed-point 40-bit number including 19 fractional bits. We scale the output of the divider to avoid fractional arithmetic. Finally, we store the result of this stage in the $L_\mathrm{flow}$ BRAMs.

4 $$\begin{aligned} L_\mathrm{flow}=\frac{1}{1+\frac{L^2_x+L^2_y}{K^2}}=\frac{K^2}{L^2_x+L^2_y+K^2}. \end{aligned}$$

### Stage 3: FED filtering

In the third stage, we combine the data from $L_\mathrm{flow}$ and $L_{t}$ BRAMs to compute the sublevels in the scale space. The AKAZE algorithm uses FED filters to generate sublevels and different octaves. The main processing part of this step is the FED cell module which requires a $3 \times 3$ window of $L_{t}$ data and a $3 \times 3$ window of $L_\mathrm{flow}$ data. To prepare the input data for the FED cell in parallel, we use two 3-row line buffers for $L_{t}$ data and $L_\mathrm{flow}$ data, respectively. We compute the output of a FED cell module according to Eq. (2).

**Fig. 8:**
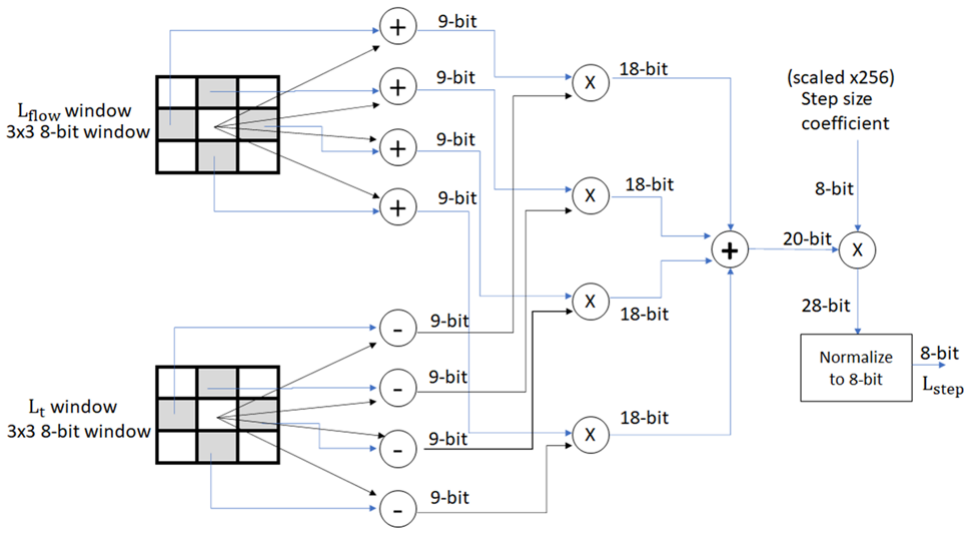
FED cell architecture

The architecture of this module is shown in Fig. 8. Each sublevel is generated by the iterative use of FED filters, with the number of FED cells required for each sublevel being different. In this stage, the FED loop is unwrapped to the maximum number of FEDs in the algorithm to achieve a pipelined architecture.

**Fig. 9:**
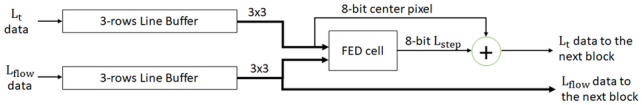
FED block architecture which contains two line buffers, an FED cell, and an adder

**Fig. 10:**
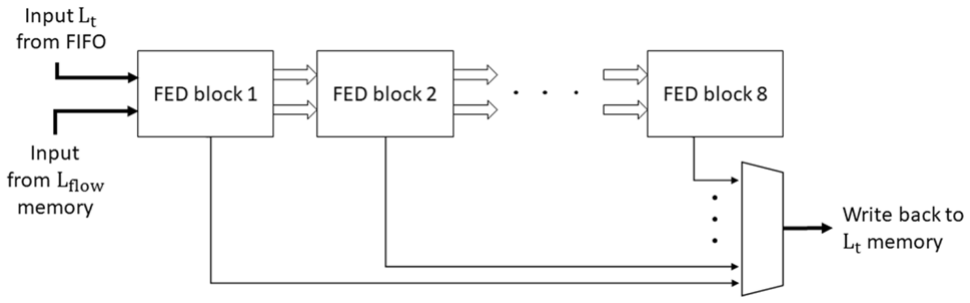
FED channel architecture which consists of 8 FED blocks

We label each package of an FED cell and two line buffers as an FED block. Figure 9 demonstrates an FED block which generates the output specified in Eq. (3). For generating the first octave, we require four of these FED blocks sequentially, which means that the output of each one is connected to the input of the next. For each sublevel, we extract the output from a specific FED block as shown in Fig. 10. A multiplexer is used to select the appropriate output based on the sublevel we are currently generating.

**Fig. 11:**
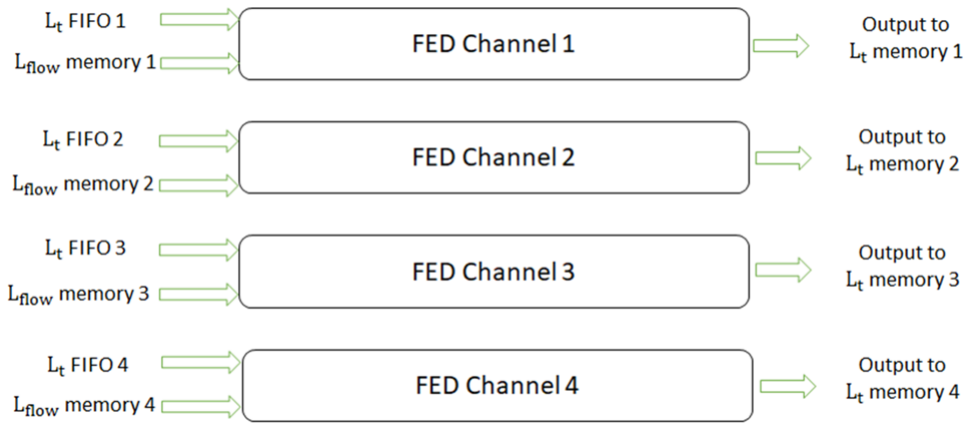
4 FED channels working in parallel

We label each group of 8 FED blocks and the multiplexer attached to them as an FED channel. Since in this design we process the data of the BRAM memories in parallel, 4 FED channels work completely in parallel. Figure 11 shows the four FED channels. We store the output of the FED channels, which are the sublevel data of the nonlinear scale space, in $L_{t}$ memory. These data overwrite the previous values of the memory which contains the data from the previous sublevel. At this stage of processing, we have the sublevel data in $L_{t}$ BRAMs. Now, the diffusivity stage can start again to generate the next $L_\mathrm{flow}$ for the next sublevel.

### Memory management unit

The main contribution in this work is represented in the memory management unit. We have two memories which are dedicated to $L_{t}$ data and $L_\mathrm{flow}$ data. The $L_{t}$ data are the sublevels of the nonlinear scale space and therefore are the output of the algorithm while $L_\mathrm{flow}$ data are computed as the required data in the middle of the processing of each sublevel. Each of the memories are divided into *n* smaller BRAMs (in this design we use $n=4$), which can be independently written or read. All of these memories are configured as dual port RAMs.

In the first stage (preprocessing) the filtered pixels of the image are written into the four BRAMs of $L_{t}$ sequentially as shown in Fig. 3. The first BRAM is filled and then, the second. This continues until all data are completely read. The algorithm then waits until the contrast factor is computed.

Then, since we have access to all of the image data in the $L_{t}$ BRAM, we can read from the four BRAMs in parallel. In the second stage of the algorithm, diffusivity channels read the data from the four $L_{t}$ BRAMs in parallel. Since four diffusivity channels are working in parallel, we can write the data into $L_\mathrm{flow}$ BRAMs in parallel as well. In our design, we use port A of the $L_\mathrm{flow}$ BRAMs to write the $L_\mathrm{flow}$ values as the outputs of the diffusivity stage. As soon as writing the data is started in the $L_\mathrm{flow}$ BRAMs, the third stage of the algorithm can start working. In the third stage, FED channels read the data from the $L_\mathrm{flow}$ BRAMs through port B and process them in parallel. When the output of this stage is ready, it will write back the results into the $L_{t}$ BRAMs through port B. The architecture of this design is illustrated in Fig. 3.

Another key element of the memory management unit is the $L_{t}$ FIFO between the second and third stages. Since both ports of each $L_{t}$ BRAM are being used, to speed up the design, we use FIFO memories to send the required $L_t$ data from the diffusivity stage to the FED stage. By using a FIFO architecture, we can synchronize the flow of the $L_{t}$ data and the $L_\mathrm{flow}$ data to have them available at the same time in the third stage.

**Fig. 12:**
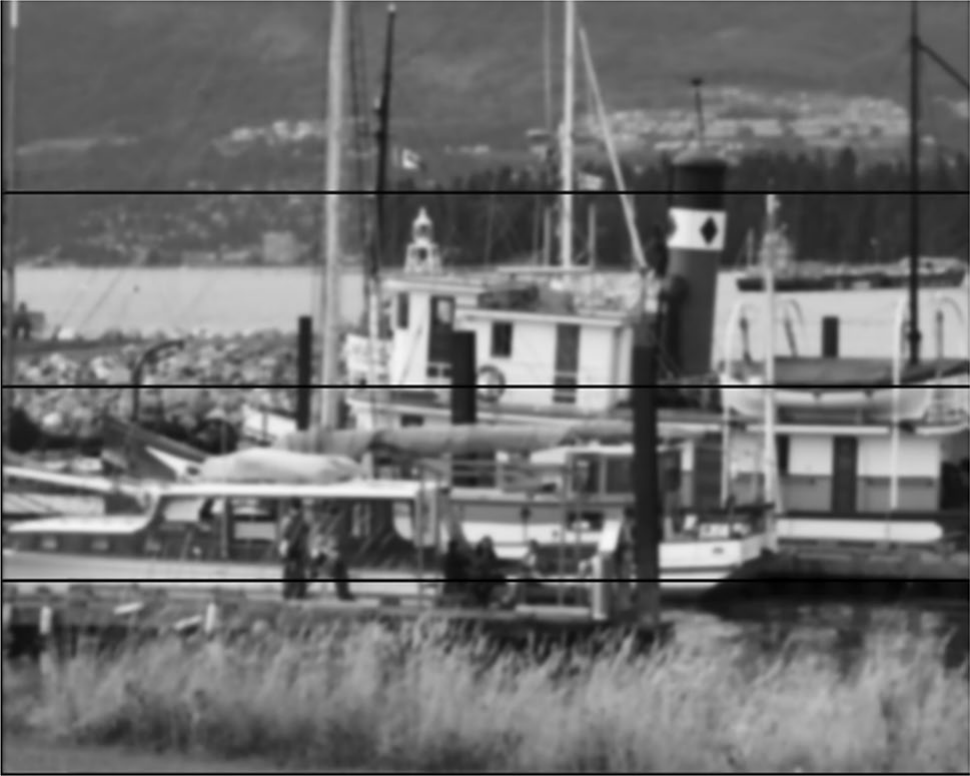
An example of the artifact from processing four sections of the image in parallel. Image from Oxford affine covariant features dataset [19]

Processing the data in each of the *n* BRAMs separately leads to some undesirable artifacts on the generated output. An example of this artifact is shown in Fig. 12 as black horizontal lines in the image. The reason for this artifact is that the first rows and the last rows of each section require the data of the adjacent rows from previous and subsequent sections, respectively. To prevent this artifact, we use a time-sharing mechanism to provide each processing channel with the required data.

To prevent the artifacts caused by the border rows in the diffusivity stage, we define three phases for processing each section. There are 4 channels of processing in the diffusivity stage. In the first phase, each channel reads the values from the last two rows of the previous section. As a result, the initial values of the line buffers will be filled with the data from the previous section of the image. In the second phase, each channel reads the data from its own corresponding section in the memory. This phase, which is the main phase of the process, utilizes most of the time of this stage. In the third phase, each channel reads two rows of the data from the next section of the image from the memory. Therefore, the channel has access to the required information from the next section. To implement this time-sharing mechanism, we add data multiplexers to the beginning of each diffusivity channel. In addition, we use finite state machines to issue the required control signals for each phase.

Since the diffusivity stage and FED stage work simultaneously, when the process in the second phase reaches the last row of a section, the first rows of the next section are already updated with the next sublevel values in the memory. Therefore, we cannot use the current data to prevent the artifact. The solution to this problem is to store the first two rows of each section in another part of the memory and use it in the third phase. We propose a “helping” memory which has the capacity of storing two rows of each section. In each iteration of the algorithm, we fill the helping memories when reading the first two rows of each section in phase two and load from the helping memories of the next section in phase three.

**Fig. 13:**
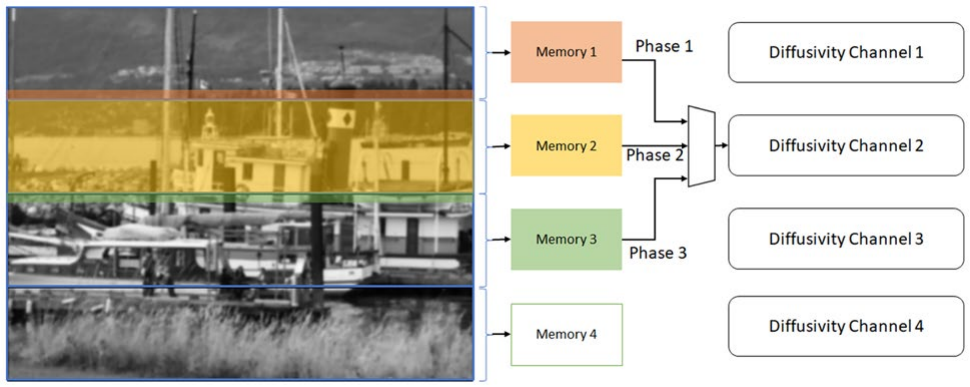
An example of selecting three phases for reading data from various sections of the memories. We show the data flow for diffusivity channel 2 as an example. In phase 1, this channel reads the data from the last two rows of the first section of the image. In phase 2, data enter channel 2 from the second section and in phase 3, diffusivity channel 2 reads the first two rows of data from the next section. Other channels have a similar data flow. Image from Oxford affine covariant features dataset [19]

Since the first section of the image does not have a previous stage, the line buffers are filled with zeros in the first phase for the first channel. Similarly, we use zeros as the input data for the last channel in phase 3 since there is no section after that. Therefore, memories 1 and 2 are connected to the diffusivity channel 1 using a multiplexer. Memories 1, 2, and 3 are connected to the diffusivity channel 2 using the second multiplexer. Memories 2, 3, 4 are connected to the diffusivity channel 3 using the third multiplexer and memories 3 and 4 are connected to the diffusivity channel 4 using the fourth multiplexer. We use the same procedure for FED channels and $L_\mathrm{flow}$ memory to prevent the artifacts. Figure 13 demonstrates the time-sharing mechanism for preventing the line artifacts in the nonlinear scale space.

### Image resizer

In the original AKAZE algorithm, after each octave is generated, the size of the image is reduced by half. In our design, the image resizer module issues the required signals to store only half of the image in the memory to resize the image. To do so, this module controls the write enable signals of the port B of $L_t$ BRAMs. When we are generating the first level of the second octave, the resizer module disables the write enable signal when the FED channels are generating the outputs of even rows and even columns. Therefore, only odd rows and columns are written into $L_{t}$ BRAM memories and the size of the image is thus reduced by half.

**Fig. 14:**
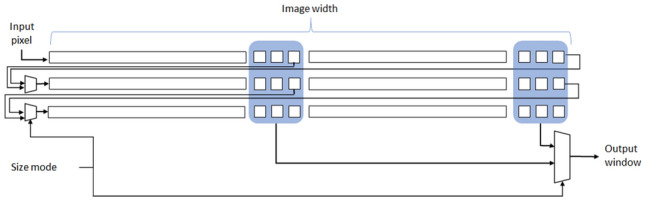
The architecture of the 3-row line buffer with multi-scale capability

After this step, all other parts of the design work with the smaller image. To do so, we design each of the line buffers in the diffusivity and FED stages to have the capability to work with two sizes. The architecture of the line buffers with three rows is shown in Fig. 14. If the line buffer has more than three rows (for example, 5 or 9 rows) the concept is the same and only the number of the registers is different.

The line buffers have two modes. In the first mode, we use the full capacity of the line buffers. The input pixels at the end of each line are written to the beginning registers of the next line. In this mode, the output window is derived from the last registers of each line. This mode is used when we are processing the first scale of the image. The second mode, which is for half scale of the image, the output of the registers in the middle of the original line buffer is sent back to the next line. Therefore, we need to use multiplexers to select the correct input for the first registers of each row. In addition, the output window is derived by the registers in the middle of the line buffer. Therefore, there is also a multiplexer to choose the appropriate window as the output of the module. All of the multiplexers in the line buffers are controlled using a size mode signal which is generated by the level controller module that contains a counter that keeps counts of the sublevels being generated.

## Timing analysis

**Fig. 15:**
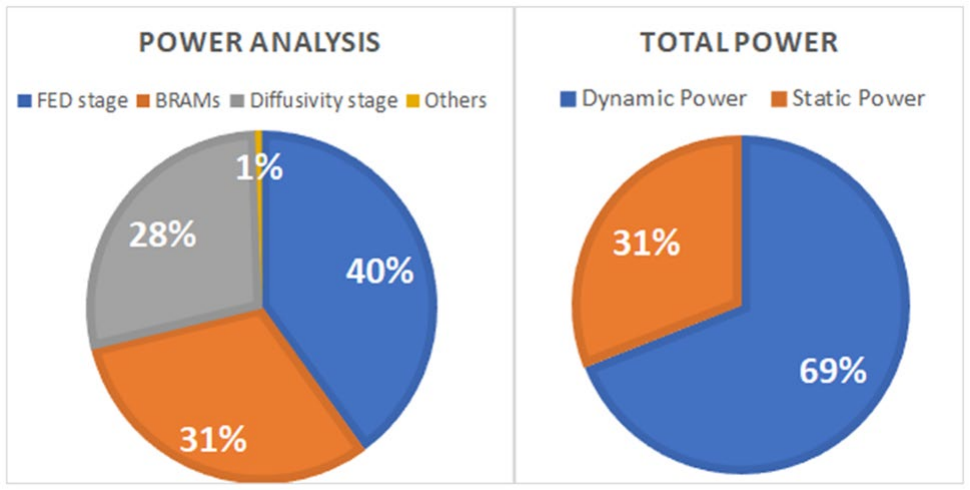
Power consumption. The left diagram shows the portion of power consumed by different stages of the algorithm. The right diagram shows the dynamic and static power consumption. Total power consumption of the design is 1095 mW

In this section, we analyze the required timing of the architecture and calculate the throughput of the design, after each line buffer is initialized. This initialization time is needed until the output of the line buffers becomes valid and we can have access to the data of multiple rows in parallel. We use zero padding to process border pixels to avoid reducing the part of the image that we are processing (Fig. 15).

In the preprocessing stage, we have a $9 \times 9$ Gaussian filter module. Therefore, we need a 9 × W  line buffer where W is the width of the image. The initialization time required for this stage is $5 \times W$ since after 5 rows of the image are read, we can have valid output from this module (other rows are initially 0s). After $5 \times W$ clock cycles, the output of the Gaussian filter is valid and after that we need $W \times H$ clock cycles to process the whole image. In this estimation we did not include the contrast factor calculation since it overlaps with filtering the data and its overhead is negligible. Therefore, the required time for preprocessing is

5 $$\begin{aligned} T_\mathrm{preprocessing}=5W+WH=W(5+H) \end{aligned}$$

The next stage of the design is the diffusivity stage. In this stage, we first have line buffers for generating a $3 \times 3$ windows as inputs for the Scharr filters. These line buffers require $2 \times W$ clock cycles for initialization and is the first phase of the time-sharing mechanism. After that, since we are processing the image in *n* different sections in parallel, we require ($W \times H)/n$ clock cycles to read and process *n* sections of the image. In addition, an initial 43 clock cycles are required for the divider module. After that, at each clock cycle, the divider generates new valid results. Hence, the number of required clock cycles for the diffusivity step is based on the image width, height, and the number of parallel sections according to:

6 $$\begin{aligned} T_\mathrm{Diffusivity}=2W+\frac{WH}{n}+43 = W(2+\frac{H}{n})+43 \end{aligned}$$

The next stage is the FED module. In this stage, similar to the diffusivity stage, we use 3-row line buffers in each FED block module. Therefore, we need $2 \times W$ for initialization of each FED block module. In addition, $W \times H/n$ clock cycles are required for reading and processing the pixels of each section of the image. Since for each sublevel we get the output from a different FED block, we do not need to wait for the data to pass all the FED blocks in an FED channel in this stage. The first octave has four sublevels. The first sublevel is the filtered image and therefore there is no need to compute the result of the FED stage for it. For the second and third sublevels, we get the outputs from the second FED block and for the fourth sublevel, we get the output from the third FED block. In the second octave, for the four sublevels of five, six, seven, and eight, we get the output from the third, fourth, fifth and sixth FED block, respectively. It is important to note that for the second octave, the size of the image is reduced to half size and therefore we use *W*/2 and *H*/2 as width and height of the image. Hence, the number of required clock cycles for this stage is:

7 $$\begin{aligned} T_\mathrm{FED}= \, & {} \left( 2W(2+2+3)+\frac{WH}{n}\right) \nonumber \\&+\left( \frac{2W}{2}(3+4+5+6)+\frac{WH}{4n}\right) \nonumber \\= & {} W\left( 32+\frac{5H}{4n}\right) \end{aligned}$$

Summing up the required clock cycles for one frame and dividing by the frequency, the total delay of our design is:

8 $$\begin{aligned} {T_\mathrm{delay}}= \, & {} \frac{1}{\mathrm{frequency}}\times \left( \frac{1.25WH}{n}+32W\right) \nonumber \\= & {} \frac{W}{\mathrm{frequency}} \times \left( \frac{1.25H}{n}+32\right) \end{aligned}$$

The important difference in our work is the parameter *n*. If we use $n=1$, the throughput of our design is similar to that of Kalms’ work [15] and the frame rate would be 98 frames per second. If we use $n=4$, which means having 4 memory sections, we can achieve 360 frames per second for the same image resolution ($1024 \times 768$) at a maximum clock frequency of 102.7 MHz (rounded off to 100 MHz in Table 2 for ease of comparison with other work) on the *Kintex*^®^ *Ultrascale*™ FPGA. This number is also confirmed by our simulation results. We can readily synthesize this design for different image resolutions for various applications.

## Experimental results

In this section, we provide the implementation results and evaluation metrics of our work and compare our results with other related work. We use the KCU105 FPGA board which contains a *Xilinx*^®^ *Kintex*^®^ *Ultrascale*™ FPGA for synthesizing our design. Results demonstrate the performance of hardware design which is synthesized and simulated using *Vivado*^®^ software.

**Fig. 16:**
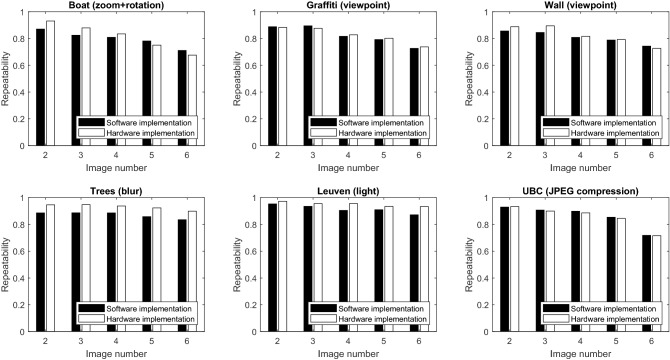
Comparison of repeatability between the software implementation and the hardware implementation based on simulation using image sets of the Oxford affine covariant features dataset [19]

Table 1 shows the resource usage of the stages of the design. In this table, LUTs are the Look up tables which are the smallest logic blocks in the FPGA. DSP represents the number of Digital Signal Processors which are the arithmetic units in the FPGAs and FF shows the number of Flip Flops which represents the number of registers used in the design. Figure 16 shows the power consumption of different stages of the design. The design consumes a total power of 1095 mW.

**Table 1:** Resource consumption of the stages of the algorithm

Algorithm stages	LUTs	Block RAMs	DSP	FF
Diffusivity stage	22935	0	0	15016
FED stage	79454	0	29	43714
Preprocess stage	9187	0	0	5378
Memory management unit	620	524	2	805

Table 2 demonstrates the overall resource usage, frequency and speed of our implementation in comparison with other work. In comparison with the work by Jiang et al. [13] our work achieves higher frame rate, even though their work does not contain the contrast factor calculation. Our frame rate is higher than that of Kalms et al. [15], while our frame size is bigger. In comparison with Li et al. [17], our resolution is higher than their work, and still we use less LUTs (but more BRAM). If we use the same resolution as their work which is $640 \times 480$, our frame rate is 862 frames per second. Based on the results of Li et al. [17], their method affects the final accuracy. Therefore, with the same image resolution, our design achieves the highest frame rate using the same frequency.

**Table 2:** Comparison of design metrics

FPGA resources	Ours	Kalms et al. [15]	Jiang et al. [13]	Li et al. [17]
FPGA/Platform	Kintex^®^ Ultrascale^™^	Zynq^®^	ASIC	Kintex^®^-7
LUT	112596	16507	–	196134
LUTRAM	72276	–	–	28068
BRAM	524	60	–	291
DSP	31	149	–	228
FF	65028	22738	–	157122
Image resolution	1280 $\times$ 720	1024 $\times$ 768	1920 $\times$ 1080	640 $\times$ 480
Frequency	100 MHz	100 MHz	200 MHz	100 MHz
Frame rate	304 fps	98 fps	127 fps	784 fps

We designed and synthesized the proposed hardware using VHDL in *Vivado*^®^ 2017 software. We also created a software model of the hardware in VHDL in MATLAB^®^ for accuracy evaluation purposes. This software model produces identical results as the hardware implementation. Since the focus of this paper is on nonlinear scale space generation, we do not need a complete matching system to compare the results. However, by adding the same key-point detector to both software implementation and the model of our hardware, we can use the repeatability metric to evaluate our design.

Other work has used different metrics to demonstrate the performance of their design. Jiang et al. [13] introduce a descriptor and report the performance of the whole system on the Oxford dataset [19]. Li et al. [17] use a self-synthesized dataset for accuracy evaluation. Kalms et al. [15] use FREAK descriptor and report the performance of the whole system which is also affected by FREAK descriptor. Since these work do not use the same metric for evaluation and the focus of our work is on nonlinear scale space generation, we decided to use repeatability [19] to show the correctness of the design. Higher repeatability implies improved performance of the feature detector which is the step after nonlinear scale space generation in an image matching system. Hence, this is an appropriate metric for demonstrating the performance of this design. This metric demonstrates how many key-points in the first image are found in the second image and is defined in Eq. (9):

9 $$\begin{aligned} \mathrm{Repeatability}=\frac{{\# {\text {of correspondences}}}}{{\# {\text {of key-points in the first image}}}} \end{aligned}$$

We use the Oxford affine covariant features dataset [19] for comparing the repeatability of the software and the hardware implementation of the AKAZE algorithm. We use MATLAB^®^ for software implementation of the algorithm. The Oxford dataset contains a variety of image sets with different transformations such as changes in rotation, scale, viewpoint, and illumination. Each set has 6 images from which the results of matching key-points of the first image with other images, are used in the evaluation. We add a Hessian detector to the nonlinear scale space images to find the key-points for evaluation. The software implementation is based on floating-point and the hardware implementation uses integer arithmetic which is scaled to improve the computations. As shown in Fig. 16, the repeatability of the hardware implementation is close to the software implementation. The small difference is due to the approximations in bit-width in hardware design. We observe that for some of images, software is better and in other images hardware can be better. Since we are focusing on the nonlinear scale space filtering, approximations in bit-width have a direct effect on the output images. It may cut off some of the details from the images in lower bits. This could result in more matches in some images depending on the image content.

## Conclusion

In this work, we propose a design for nonlinear scale space generation for the AKAZE algorithm. Using nonlinear scale space for image matching leads to a higher accuracy but requires more computations.

The first contribution of this work is based on the idea to take advantage of the nature of the AKAZE algorithm which uses two passes through the image. This gives us an opportunity to use four parallel channels to generate a nonlinear scale space. In previous implementations of the AKAZE algorithm [15], the image data are read from an external memory in the first step to filter the image and compute the contrast factor. Then, the result is written back to the memory so that it can be read again for the next stage. We take advantage of this fact that in the first step, the image is read once from the external memory and we can have access to different sections of the image if we store it on chip in separate memories. Therefore, we design the memory management unit to store the image in 4 separate BRAMs so that we can generate the sublevels of each section of the image in parallel. This, in addition to the fully pipelined architecture of each stage of the algorithm, leads to a noticeable speed up in our design.

The second contribution of this work is the architecture we propose for the second octave line buffers which uses the same data path as the first octave, but in a different scale. For this part, we introduce multi-scale line buffers which have several output windows for parallelizing the image input at different scales. Using traditional architecture results in consuming twice the number of the line buffer registers because each scale requires its own line buffers. However, by changing the architecture of the line buffers, we use the same hardware resources for both scales.

The third contribution of this work is the time-sharing mechanism in the memory management unit which provides the opportunity to process different sections of the image in parallel without having artifacts in the image. We introduce the time-sharing mechanism for this stage which has three phases in Sects. 4 and 5. By using this architecture, we can process multiple sections of the image which are stored in different memories in parallel and provide the border pixel values to all processing channels to prevent artifact in the images. With these contributions, we achieve 304 frames per second for $1280 \times 768$ image resolution. We demonstrate that the approximations proposed in our hardware implementation do not have a significant negative impact on the repeatability of the algorithm based on the results in Fig. 16.

Possible future avenues of investigation could include considering other diffusion algorithms to assess their suitability for hardware implementation and considering different detectors and descriptors that can be added to the current architecture, following the parallel channel processing concept.
